# Do CRISPR Germline Ethics Statements Cut It?

**DOI:** 10.1089/crispr.2017.0024

**Published:** 2018-04-01

**Authors:** Carolyn Brokowski

**Affiliations:** Department of Emergency Medicine, Yale School of Medicine, New Haven, Connecticut.

## Abstract

The extraordinary wave of genomic-engineering innovation, driven by CRISPR-Cas9, has sparked worldwide scientific and ethical uncertainty. Great concern has arisen across the globe about whether heritable genome editing should be permissible in humans—that is, whether it is morally acceptable to modify genomic material such that the “edit” is transferable to future generations. Here I examine 61 ethics statements released by the international community within the past 3 years about this controversial issue and consider the statements' overarching positions and limitations. Despite their inability to fully address all important considerations, many of the statements may advance debate and national and international law and public policy.

## Introduction

In February 2017, the United States National Academies of Sciences, Engineering, and Medicine (NASEM) Committee on Human Gene Editing published an expansive report^1^ reviewing scientific, ethical (moral), and legal concerns about the astonishing rise of genomic engineering technology. The NASEM committee, chaired by Massachusetts Institute of Technology/Howard Hughes Medical Institute molecular biologist Rick Hynes and University of Wisconsin bioethicist Alta Charo, assembled 22 international experts from the fields of biomedicine, law, and bioethics. The report's chief conclusion, surprising to some, was that heritable genome editing^*^—the modification of the germline with the aim of generating a new human being who could therefore transfer the genomic change to future generations—should be impermissible now^2^ but eventually could be justified for certain medical indications. However, the NASEM committee did not sanction the use of CRISPR^†^ for any form of enhancement. Currently, it is unlawful for U.S. federal funds to be used to create, destroy, or modify human embryos to include heritable genetic changes for research purposes.^3–5^ Yet the NASEM report's conclusion implies that once safety risks are better understood, then clinical trials conceivably could commence.

Safety, risk/benefit, and efficacy concerns are familiar territory in the context of somatic genome editing—the modification of nonreproductive cells such as cardiomyocytes, monocytes, and osteoblasts. Human gene therapy, for example, which arose during the 1980s and 1990s, involves the application of nucleic acid sequences or genetically engineered organisms for investigational and/or therapeutic purposes. In 1999, Jesse Gelsinger, an 18-year-old diagnosed with ornithine transcarbamylase deficiency, a rare recessive X-linked disorder, died in a phase-1 dose-escalation clinical trial. Though controversial, many attribute his death to a massive immune response against the high-dose adenoviral vectors administered during the research. Today the risks in gene therapy trials are better understood,^6,7^ although safety, efficacy, and other ethical matters remain unresolved. Still, many gene therapy trials are underway, including some using more traditional forms of gene editing such as zinc finger nucleases.^8^ The rapid emergence of CRISPR gene editing technology, however, has significantly intensified the need for scientific, medical, and ethical evaluation of the potential benefits and risks of gene editing. Given the pace of scientific discovery in this field and early reports on the deployment of CRISPR gene editing in human embryos, it is not too soon to ask whether it is morally acceptable to modify the human germline using this approach. As of March 2018, three groups have published studies involving human germline editing.^9–11^ Two reports arose from groups in China and were followed by a major study from Shoukrat Mitalipov and colleagues in *Nature* in August 2017, which reported the successful correction of a defective gene in human embryos.^9^

**FIG. 1.**
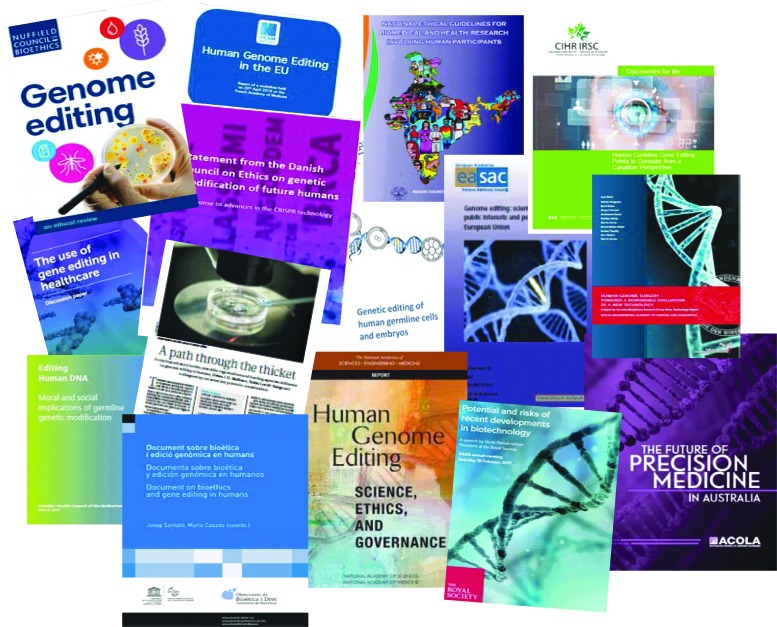
Cover Story: More than 60 official reports and statements about the ethics of germline editing have been published within the past three years.

Not surprisingly, the international community's views about gene editing for clinical purposes, and especially the possibility of germline editing, vary enormously. At least 61 ethics reports and statements^‡^ have been crafted by more than 50 countries and organizations over the past 3 years (Table 1^1,12–73^; Fig. 1). Statements have been published by U.S. and international scientific societies such as the American and European Societies of Human Genetics, the European Society of Human Reproduction and Embryology, and the International Society for Stem Cell Research; bioethics organizations including the Nuffield Council in the United Kingdom, the Danish Council on Ethics, and the International Bioethics Committee of the United Nations Educational, Scientific and Cultural Organization; industry groups and organizations including the Biotechnology Innovation Organization and various genome-editing biotech companies; and political groups such as the 2015 White House.

**Table 1.** **International reports on human heritable germline editing: 2015–2018**

*Region(s)*	*Report No.*	*Year*	*Group*	*Distinction between preclinical vs. clinical research*	*Moratorium^a^*	*Should be impermissible currently*	*Mechanical/technical concerns^b^*	*Safety concerns*	*Enhancement should be impermissible currently*	*Difficulties with informed consent*	*Public input encouraged*	*Societal consensus encouraged*
Australia	1	2018	Australian Council of Learned Academies^12^	+	0	0	+	+	0	0	+	0
Canada	2	2018	Centre of Genomics and Policy, McGill University and Génome Québec Innovation Centre^13^	+	0	0	+	+	0	0	+	0
	3	2016	Canadian Institutes of Health Research^14^	+	0	0	+	+	0	0	+	0
Denmark	4	2016	Danish Council on Ethics^15^	+	0	+	+	+	+	+	0	0
France	5	2017	Alliance VITA^16^	+	0	+	+	+	+	+	0	0
	6	2016	Académie Nationale de Médecine^17^	+	0	+	+	+	0	0	+	0
	7	2016	Institut National de la Santé et de la Recherche Médicale^18^	+	0	+	+	+	0	0	+	0
Germany	8	2017	German Ethics Council^19^	+	0	0	+	+	+	0	+	0
	9	2017	Leopoldina, Nationale Akademie der Wissenschaften^20^	+	0	+	0	+	+	0	0	0
	10	2015	Berlin-Brandenburg Academy of Sciences and Humanities^21^	+	+	+	+	+	0	+	+	0
	11	2015	German Stem Cell Network^22^	+	+	+	+	+	0	0	+	0
Greece	12	2016	Hellenic National Bioethics Commission^23^	+	0	+	+	+	0	0	+	0
India	13	2017	India Council of Medical Research^24^	+	0	+	+	+	0	0	+	0
Japan	14	2017	Science Council of Japan^25^	+	0	+	+	+	0	0	+	0
Multinational	15	2018	European Society of Human Genetics & European Society of Human Reproduction and Embryology^26,27^	+	0	+	+	+	0	0	+	0
	16	2017	American Society of Human Genetics et al.^28^	+	0	+	+	+	0	+	+	0
	17	2017	Chneiweiss et al.^29^	+	-	0	+	+	0	0	+	0
	18	2017	Council of Europe^30^	+	0	+	**+**	**+**	+	+	**+**	0
	19	2017	European Academies Science Advisory Council^31^	+	0	+	**+**	**+**	0	0	**+**	**+**
	20	2017	European Society of Human Genetics^32^	+	0	0	+	+	0	0	+	0
	21	2017	Federation of European Academies of Medicine^33^	+	-	+	**+**	**+**	**+**	0	**+**	0
	22	2016	European Group on Ethics in Science and New Technologies^34^	0	+	+	**+**	**+**	0	0	**+**	0
	23	2016	Federation of European Academies of Medicine, UK Academy of Medical Sciences, & French Academy of Medicine Workshop^35^	+	0	0	**+**	**+**	0	0	**+**	0
	24	2016	Latin America Workshop^36^	+	−	0	**+**	**+**	0	0	**+**	**+**
	25	2015	American Society of Gene & Cell Therapy and Japan Society of Gene Therapy^d,37^	+	0	+	**+**	**+**	**+**	0	**+**	**+**
	26	2015	European Society of Gene & Cell Therapy^38^	0	+	+	0	**+**	0	0	0	0
	27	2015	Hinxton Group^39^	+	0	+	**+**	**+**	0	0	**+**	0
	28	2015	Hinxton Steering Committee^40^	+	0	0	**+**	**+**	0	0	**+**	0
	29	2015	Intellia Therapeutics & CRISPR Therapeutics^41^	+	0	+	**+**	**+**	0	0	**+**	0
	30	2015	International Bioethics Committee, United Nations Educational, Scientific and Cultural Organization^42^	+	+	+	**+**	**+**	0	0	**+**	0
	31	2015	International Institute of Advanced Studies^43^	+	0	0	**+**	**+**	0	+	0	0
	32	2015	International Society for Stem Cell Research^44^	+	+	+	+	**+**	0	0	**+**	0
	33	2015	International Summit on Human Genome Editing^45^	+	0	+	**+**	**+**	0	0	**+**	+
Netherlands	34	2017	Commission on Genetic Modification and Health Council of the Netherlands^46^	+	0	−	**+**	**+**	+	0	**+**	0
	35	2016	Royal Netherlands Academy of Arts and Sciences^47^	+	0	+	**+**	+	0	0	**+**	+
New Zealand	36	2017	Royal Society of New Zealand^48,49^	+	0	0	+	+	0	+	**+**	0
Norway	37	2016	Norwegian Biotechnology Advisory Board^50^	+	0	+	+	+	0	+	0	0
Spain	38	2016	Bioethics and Law Observatory of the University of Barcelona^51^	+	0	−	+	+	+	0	+	0
United Kingdom	39	2017	Academy of Medical Sciences^52^ *See report numbers 45 & 46*	**+**	0	−	**+**	**+**	0	0	**+**	0
	40	2017	Genetic Alliance UK & Progress Educational Trust^53^	+	0	0	+	+	+	0	+	0
	41	2017	House of Commons Science and Technology Committee^54^	+	0	0	+	+	0	+	+	0
	42	2017	Royal Society^55^	+	0	0	0	+	0	+	+	0
	43	2016	Cambridge Public Policy SRI^56^	+	0	0	+	+	0	+	+	0
	44	2016	Muslim Council of Britain^57^ *See also report number 45*	+	+	+	0	0	+	0	+	0
	45	2016	Nuffield Council^58^	+	0	0	+	+	0	0	+	0
	46	2015	UK Joint Statement^59^	+	0	0	0	0	0	0	+	0
United States	47	2017	Alliance for Regenerative Medicine^60^	0	0	+	0	+	0	0	0	0
	48	2017	American College of Medical Genetics and Genomics^61^	+	0	+	**+**	**+**	0	0	**+**	0
	49	2017	Biotechnology Innovation Organization^62^	+	0	+	0	+	+	0	+	0
	50	2017	Merck^63^	+	0	+	**+**	+	0	0	0	0
	51	2017	National Academies of Sciences, Engineering, and Medicine^1^	+	0	+	**+**	+	0	+	**+**	0
	52	2016	California Institute for Regenerative Medicine^c64^	+	0	+	**+**	+	0	0	+	−
	53	2016	National Society for Genetic Counselors^65^	+	0	+	+	**+**	0	0	0	0
	54	2015	American Society for Investigative Pathology^66^	0	+	+	**+**	**+**	0	0	**+**	0
	55	2015	Baltimore et al.^67^	+	0	+	**+**	**+**	0	0	**+**	0
	56	2015	Center for Genetics and Society^68^	0	0	+	**+**	**+**	+	0	**+**	0
	57	2015	Editas Medicine^69^	0	0	0	0	0	0	0	+	0
	58	2015	National Institutes of Health^70^	+	0	+	0	+	0	+	0	0
	59	2015	Nature Institute^71^	+	0	+	+	+	0	0	0	0
	60	2015	Society for Developmental Biology^72^	+	+	+	+	+	**+**	0	0	0
	61	2015	White House^73^	+	0	+	0	0	**+**	0	0	0

+, Expressly stated; −, expressly denied; 0, not expressly addressed or ambiguous.

^a^ Temporary prohibition of any activity.

^b^ Relating to whether CRISPR-Cas9 functions such that it produces expected biological outcomes without confounding factors such as inaccurate editing (off- and on-target effects), incomplete mosaicism, efficiency challenges, and interference from unanticipated and/or poorly understood factors (e.g., epigenetic, immune, and environmental events, pleiotropy, and penetrance).

^c^ Draft recommendations.

^d^ Currently named the Japan Society of Gene and Cell Therapy.

Although these statements vary considerably in both length and depth of analysis, they provide a large body of scholarship in which to frame and discuss the medical and moral permissibility of heritable genome engineering. Most statements were produced by organizations from Europe and the United States, though groups from Canada, Latin America, New Zealand, Japan, China, Australia, and other international conglomerates also contributed (Table 1). From a bioethics or legal perspective, many of these reports are limited. However, some of the questions raised and debated provide a preliminary basis for addressing key critical issues and advancing international law and public policy in this arena.

## Take Your Positions

**FIG. 2.**
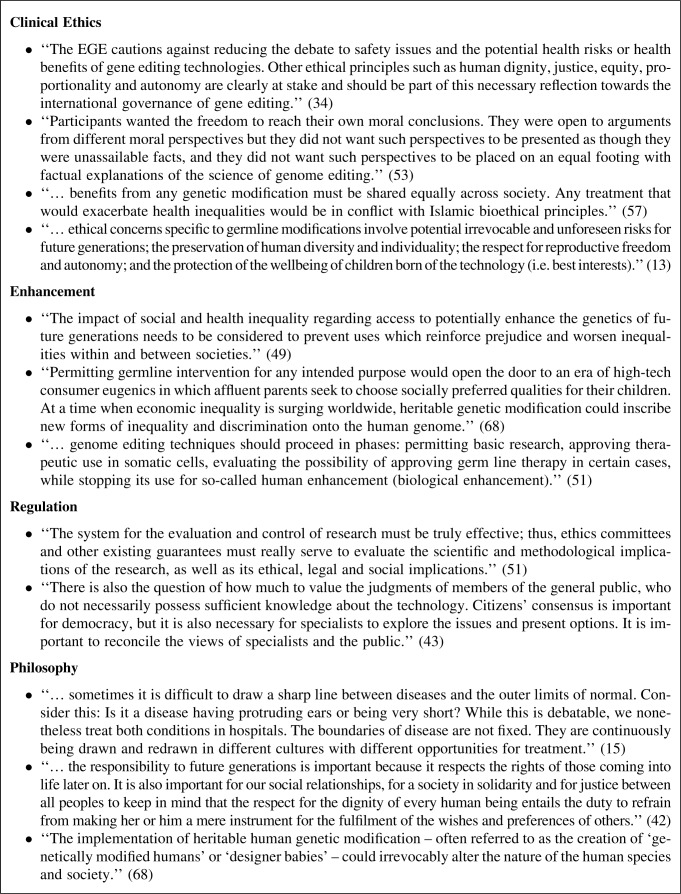
Bioethics considerations. Extracts from some selected reports on genome editing illustrate a diversity of opinions on some key bioethical issues.

**FIG. 3.**
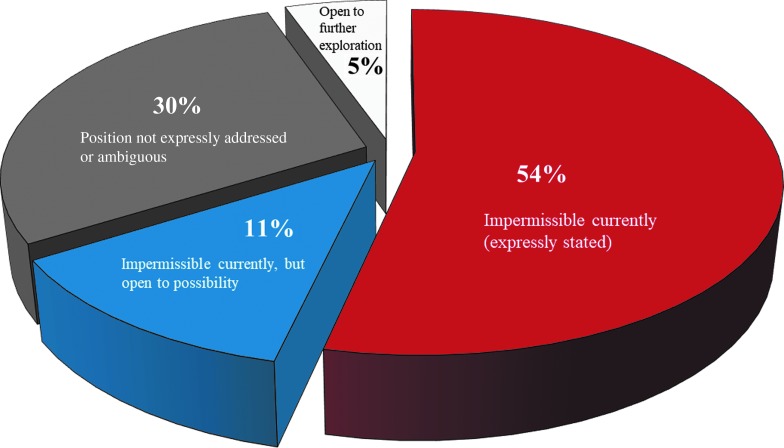
Opinions on the moral permissibility of heritable genome editing. This pie chart displays the views of 61 ethics reports on germline editing. The views represented are not logically exhaustive. The majority (54%) expressly considered germline editing impermissible at the current time.^16–18,20–22,24,25,28,30,31,33,34,37,38,41,42,44,50,57,60–68,70–73^ A further 11% also consider germline editing impermissible currently, but are expressly open to the possibility of allowing it under certain conditions.^1,15,23,26,39,45,47^ In 30% of cases, the position is not expressly addressed or is ambiguous.^12–14,19,29,32,35,36,40,43,48,49,53–56,58,59,69^ And 5% of the reports state an openness to further exploration.^46,51,52^

Statements' positions range widely—from being direct, pithy, and practical to expansive, indeterminate, nuanced, and philosophical. They also capture a variety of important issues (Fig. 2). Few groups were willing to go as far as the NASEM Committee in tentatively supporting germline editing, even under certain specified conditions. Most statements were expressly against heritable genome editing at the current time (Table 1; Fig. 3).^1,15–18,20–26,28,30,31,33,34,37–39,41,42,44,45,47,50,57,60–68,70–73^ Some favor a form of moratorium—ranging from broadly prohibiting “gene editing of human embryos or gametes which would result in the modification of the human genome”^34^ to more narrowly prohibiting “attempts to apply nuclear genome editing of the human germ line in clinical practice.”^44^ Accordingly, various categories of risk outweigh any potential benefits for now. Overall, much of the international community seems reluctant to proceed with heritable germline editing.

A common concern is that editing might pose technical/mechanical obstacles, leading indefinitely to safety risks in the modified organism and future progeny.^1,12–19,21–37,39–54,56,58,61,63–68,71,72^ Obstacles might include inaccurate editing (off- and on-target effects), incomplete editing (mosaicism), efficiency challenges (success rate), and interference from unexpected and/or poorly understood factors (e.g., epigenetic, immune, and environmental events; pleiotropy; and penetrance) resulting in unintended consequences.

Further, in a joint position statement, the American Society of Gene & Cell Therapy and the Japan Society of Gene Therapy^§^ noted that “[t]he requirement that the results of an experiment be susceptible to analysis and characterization before further applications are undertaken cannot be met with human germ-line modification with current methods, because the results of any such manipulation could not be analyzed or understood for decades or generations—a situation incompatible with ethical imperatives and with the scientific method.”^37^ Other concerns included the potential return of eugenics, human enhancement, and the exacerbation of social inequalities, along with a purported lack of “compelling medical rationale” justifying such interventions. Additionally, difficulties with obtaining informed consent,^1,15,16,21,28,30,43,48–50,54–56,70^ given the complexity surrounding the status of the human embryo and the potential effects lasting into numerous future generations, were highlighted. Many also point out that national and international laws already prohibit such modifications.

Several other groups assume more moderate positions. For instance, the 2017 joint report issued by the Netherlands Commission on Genetic Modification and the Health Council of the Netherlands maintains that, due to the limited knowledge about risk and possible clinical applications and benefits at this time, it is “not possible to come to a clear and definite conclusion about the acceptability of germline modification, but it is possible to investigate the conditions under which clinical applications of germline modification could be practised.”^46^ Therapeutic applications of germline modification could be permissible, and the report states that healthcare providers might even have a *moral obligation* to make available this option, if safety and efficacy concerns are alleviated.

Despite its opposition to heritable germline editing at the current time, the NASEM report reached a similar conditional conclusion,^1^ noting that trials would be acceptable if technical challenges of the research were resolved, the risk/benefit ratio of the proposed research were reasonable, and a “robust and effective regulatory framework” were established that would include the following:

(1) No reasonable alternatives to the trial exist;
(2) The goal of the research is to prevent a serious disease or condition;
(3) The research focuses only on editing genes that seem to predispose or cause the disease or condition;
(4) Gene conversion is limited to only those versions associated with ordinary health and that are unlikely to cause adverse effects;
(5) Credible preclinical and clinical health risk data are available;
(6) Institutions establish both ongoing monitoring of the health and safety of clinical research participants;
(7) Long-term follow-up plans are defined and implemented;
(8) Transparency and privacy protections are in place;
(9) Societal risks are controlled; and
(10) Mechanisms inhibit extension to uses other than preventing a serious disease or condition.

The biggest international gathering so far on the subjects of CRISPR and germline editing took place in Washington, DC in December 2015. NASEM, the Chinese Academy of Sciences, and the U.K.'s Royal Society co-hosted the International Summit on Human Gene Editing, which was co-organized by Jennifer Doudna and chaired by David Baltimore. One of the major conclusions of this meeting was that allowing heritable genome editing would be “irresponsible” unless and until more were known about safety, risks, benefits, and efficacy and “broad societal consensus” were achieved.^45^ Since then, however, as noted by the German Ethics Council, there seems to have been a subtle, though important, shift in opinion about the permissibility of heritable genome editing— from “impermissible as long as risks have not been determined” to “permissible if risks are accurately determined.”^19^

Finally, although recognizing potential difficulties, a statement from the U.K.'s Academy of Medical Sciences, written in response to the Nuffield Council on Bioethics' report,^58^ is one of the few documents expressly favoring the use of this application in the future, “provided [its] introduction is based on a strong evidence base, is in line with societal values, and is informed and supported by active engagement with patients and the public.”^52^ Despite its encouraging spirit, the statement cautions that heritable applications must be crafted according to “societal values, and be supported by active engagement with patients and the public to effectively communicate the conditions in which genome editing can, and cannot, be helpful.”^52^

## Limitations, Utility, and Future Directions

The international community's fast response to calls for broad discussion^67^ about germline editing is laudable. Yet for all of the earnest deliberations and valuable reports issued since 2015, there are many nagging limitations. Some statements offer conclusions but lack significant support. The Alliance for Regenerative Medicine, for instance, notes that “Patients will benefit more immediately from resources being directed towards somatic applications of the technologies at this time, as most genetic diseases manifest in and can be treated in somatic, not germline, cells.”^60^ It goes on to conclude that “heritable germline editing is not ready to be tried in humans.” Yet widespread experimentation would be required to determine whether somatic or germline editing would yield more value. As many countries outlaw or at least fail to fund such research, there is simply a dearth of robust, reliable data to ascertain potential benefits and risks of germline editing—thereby confounding the ability of the moral permissibility of experimentation.

Second, sometimes purported justifications are questionable. For instance, National Institutes of Health (NIH) Director Francis Collins is staunchly opposed to germline editing in any form. He cites as problematic “unquantifiable safety issues, ethical issues presented by altering the germline in a way that affects the next generation without their consent, and a current lack of compelling medical applications justifying the use of CRISPR/Cas9 in embryos.”^70^ Should “unquantifiable safety issues” prohibit U.S. federal funding, by the NIH and other federal agencies, of all heritable germline clinical trials? The purpose of many trials, after all, is to *assess* safety. Does not the promotion of procreative liberty, through correcting genetic defects in potentially unhealthy embryos,^74^ count as a “compelling medical application”? Ultimately, Collins might be correct in his conclusion, but additional, specific analysis would help to illuminate and justify this opposition. Further, why concede that consent complexities and alleged lack of compelling medical justification should trump the potential benefits of heritable genome experimentation, such as potentially promoting the health of defective embryos and facilitating procreative liberty? In addition, the NIH director cites “existing legislative and regulatory prohibitions” against heritable genome editing. Yet even if there are (or were) good reasons for this legislation, the justificatory weight of the laws as presented is question begging.

Third, despite the wealth of topics about heritable genome editing considered in these 60+ reports, important questions arise about how to move forward. For example, even the NASEM report does not expressly show why or how the conditions under which heritable genome editing might be justified are or should be legitimate: Why should the presence of reasonable alternatives preclude someone's decision to edit the germline of his or her gametes and/or embryo? Can trials really focus *only* on editing genes that seem to predispose or cause the disease or condition? Why not allow medical or nonmedical enhancement as a form of free speech?^75^ How might long-term follow up be possible, given the difficulty of tracking individuals throughout the lifespan? What does it mean to control societal risks, and who, if anyone, would be accountable if problems were to arise? Which mechanisms might effectively prevent the extension to uses other than preventing a serious disease or condition? And who should pay for them? That part of the NASEM's mission is to “guide the development of federal laws and regulations, improve the effectiveness of government programs, shape the direction of research fields, and inform public knowledge and dialogue”^76^ underscores the importance of further attention to, and justification for, these considerations.

Despite their value in raising questions and generating dialogue, it is unlikely that any single ethics report or position statement – now or in the future – could address *all* critical issues raised by heritable genome editing technology (Table 1). Nonetheless, if nations or groups hold strong reasons to promote, stymie, or prohibit certain areas of this research, then authoritative ethics statements could serve as the base upon which to craft national and international law and public policy. The Belmont Report,^77^ Declaration of Helsinki,^78^ and Nuremberg Code^79^ inspired the development and evolution of federal regulations governing the involvement of human subjects in research in the United States. Heritable genome ethics statements might serve as the foundation upon which to update the first two of these important documents and might be employed to inspire additional international laws. The proliferation of dozens of ethics statements seems like a reasonable first step toward solidifying and formalizing the global community's concerns.

Yet given the rapid pace of advancement in this field, it is essential that the discussion advance broadly to explain why certain conclusions could be justified and optimal at this time and how, if at all, they might advance international policymaking and law. The Nuffield Council on Bioethics' first report in 2016 reviewed ethical issues in genome editing,^58^ and later in 2018 the Council will publish another report making recommendations to inform policy and practice. These recommendations will be welcomed but will settle upon a voluminous and often contradictory series of statements gathered from all corners of the globe.

In a recent commentary published in *Nature*, Janasaoff and Hurlburt claim that the global conversation about heritable genome editing has fallen short of the “cosmopolitan conversation” that is required.^80^ Decision makers about how novel technologies will be used typically split into two camps: pioneering scientists and experts who study how such innovation might disrupt social norms, with little communication between the two camps.^80^ As a remedy, Janasaoff and Hurlburt advocate for a new foundational platform—a “global observatory for gene editing”— upon which to engage multiple stakeholders. Deliberations by this observatory would be driven not by scientific research agendas but by the values and priorities of society. The group would consist of “an international network of scholars and organizations similar to those established for human rights and climate change. The network would be dedicated to gathering information from dispersed sources, bringing to the fore perspectives that are often overlooked, and promoting exchange across disciplinary and cultural divides.”^80^

Many in the international scientific community (and others) have already called for increased public input about moral considerations in heritable genome editing,^1,12–14,17–19,21–37,39–42,44–49,51–59,61,62,64,66–69^ demonstrating sincere interest and openness to views from outside disciplines. Yet even if the observatory for gene editing proves to be an effective foundation upon which to guide the global community, important questions remain: What does “societal consensus entail,” and is it possible and/or desirable? What influence would the observatory's decisions have, and why? Should its decisions be permitted to trump scientific innovation? If so, by what authority? Even if its instantiation would be optimal, the observatory raises just as many, if not more, questions as the statements.

## Notes

^*^ Techniques involving mitochondrial replacement therapy are beyond the scope of this discussion, despite potential for overlapping medical ethics' concerns.

^†^ ***C****lustered* ***R****egularly* ***I****nterspaced* ***S****hort* ***P****alindromic* ***R****epeats.*

^‡^ The selection of reports and statements presented herein is not exhaustive but does represent a sizeable sample of those published to date.

^§^ This group is now entitled the “Japan Society of Gene and Cell Therapy.”
